# Amyand’s Hernia: A Radiological Solution of a Surgical Dilemma

**DOI:** 10.7759/cureus.33983

**Published:** 2023-01-19

**Authors:** Nitish Raj, Brian T Andrews, Raja Sood, Iqra Saani, Michael Conroy

**Affiliations:** 1 Clinical Radiology, University Hospitals Plymouth NHS Trust, Plymouth, GBR; 2 General Surgery, Medway NHS Foundation Trust, Medway, GBR; 3 Internal Medicine, Epsom and St Helier University Hospitals NHS Trust, London, GBR; 4 Internal Medicine, Medway NHS Foundation Trust, Medway, GBR; 5 Urology, Mid and South Essex NHS Foundation Trust, Southend-on-Sea, GBR

**Keywords:** appendix, computerised tomography scan, amyand's hernia, inguinal hernia, acute appendicitis

## Abstract

Amyand’s hernia is a rare condition whereby the appendix lies within the sac of an inguinal hernia; rarer still, the appendix can become inflamed (acute appendicitis) and is frequently misdiagnosed as a strangulated inguinal hernia. We report a case of Amyand’s hernia complicated with acute appendicitis. In this case an accurate preoperative diagnosis was provided by a preoperative Computerised Tomography (CT) scan, permitting planning of treatment by a laparoscopic approach.

## Introduction

Amyand’s hernia is defined as an inguinal hernial sac that contains an appendix, which may subsequently become inflamed. Claudius Amyand (c. 1660 - 6^th^ July 1740) described this condition on 6^th^ December 1735; having operated on an 11-year-old boy presenting with an inguinal abscess. At operation the cause of the abscess was found to be an encrusted pin that had perforated the wall of an appendix incarcerated within an inguinal hernia sac [1-4].

Amyand’s hernia is a relatively rare condition, accounting for 0.5-1% of all abdominal wall hernias [5], rarer still is the combination of acute appendicitis complicating Amyand’s hernia since it accounts for only 0.07-0.13% cases of acute appendicitis. The combined rarity of these events makes a preoperative diagnosis challenging; and when it does occur, it is frequently misdiagnosed as a strangulated inguinal hernia [2]. It can also be confused with peritonitis, should the intraperitoneal fluid collect in an inguinal hernia sac or a patent processus vaginalis. Either CT or ultrasound scanning can be helpful in making a definitive preoperative diagnosis [6,7].

We present a case of Amyand’s hernia complicated by appendiceal gangrene in which CT scanning led us to an accurate preoperative diagnosis, and hence treatment of the condition by a laparoscopic approach.

## Case presentation

A 76-year-old male was admitted with a 16-hour history of central abdominal pain, nausea and severe continuous vomiting. He described the pain as gradual in onset, continuous, limited to the periumbilical region (no history of migration), severe in intensity and exacerbated by movement. There was no past history of abdominal surgery. He had a 90-pack-year smoking history, consumed six units of alcohol in a week, and his social and family history was unremarkable. He did not have any previous known medical conditions.

On examination, he had tachycardia (pulse rate 109 beats per minute); other vital signs were normal. Examination revealed a mildly distended abdomen with generalised guarding and signs of generalised peritoneal irritation in all four quadrants associated with a reduction in bowel sounds. No palpable groin swelling or cough impulse was evident. Raised blood inflammatory markers were obtained as shown in Table 1.

**Table 1:** Laboratory results CRP: C-Reactive Protein, L: Litre, mg: milligram, IU: International Unit, mmol: millimoles

Test	Result	Normal ranges
Total White Blood Cell count	16.1 x10^9^/L	4.0-11.0 x10^9^/L
Neutrophil count	14.6 x10^9^/L	2.0-7.0 x10^9^/L
CRP	66.3 mg/L	0.0-5.0 mg/L
Serum amylase	338 IU/L	28-140 IU/L
Venous lactate	2.4 mmol/L	0.5-1 mmol/L

With indistinct clinical picture and biochemical markers there was a diagnostic dilemma, and a range of differential diagnoses was made including pancreatitis, bowel perforation, gallbladder perforation, bowel obstruction and bowel ischemia. Groin hernia was not included in the differential diagnosis due to lack of palpable groin swelling or cough impulse. Abdominal and chest radiographs failed to demonstrate any evidence of perforation or bowel dilatation.

**Figure 1:**
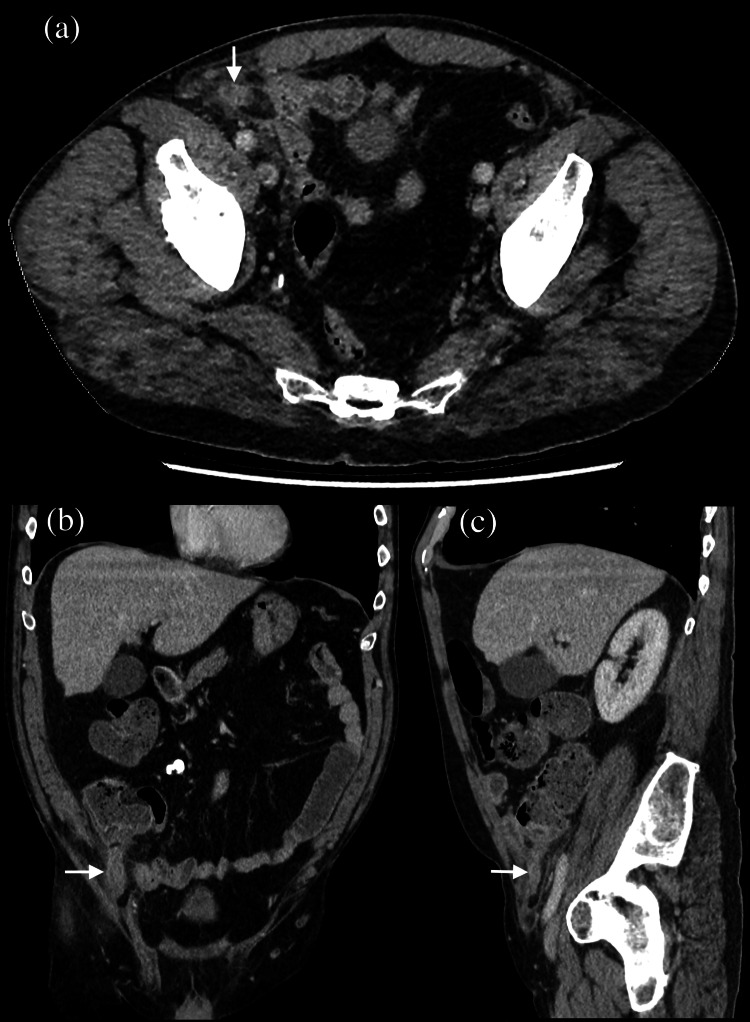
Computerised Tomography (CT) scan of abdomen and pelvis, (a) axial, (b) coronal and (c) sagittal sections in portal venous phase demonstrates terminal ileum, cecum and tip of appendix (white arrows) entering the right inguinal canal and thickened wall of the appendix with periappendiceal fat stranding.

The patient subsequently underwent contrast-enhanced CT scan of the abdomen and pelvis. This revealed a thick-walled appendix (diameter 1.5cm), showing mild irregularity of the wall, the tip of the appendix was seen entering the right inguinal canal, and was associated with significant periappendiceal fat stranding and minimal free fluid in the pelvis. Proximal small bowel loops and caecum were mildly distended; no other abnormalities were noted (Figure 1).

**Figure 2:**
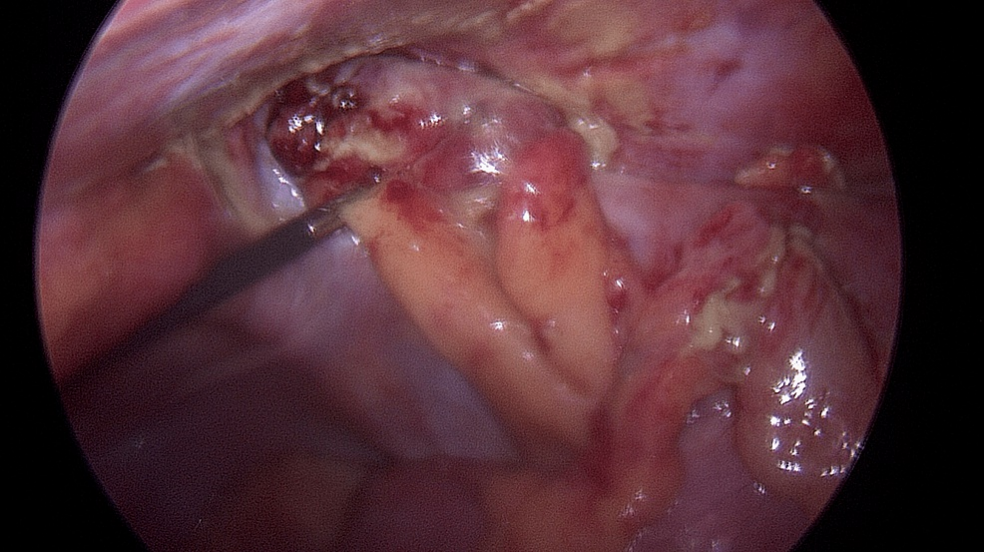
Laparoscopic view demonstrates the tip of the appendix within the right inguinal hernia sac and surrounding abscess.

A diagnosis of Amyand’s hernia with appendicitis was made and the patient was commenced on intravenous fluids, intravenous antibiotics amoxicillin/clavulanic acid (Augmentin 1.2g; Ibigen Srl, Aprilia, Italy) and analgesics. The patient was taken to the emergency surgical theatre on the same day of admission. At laparoscopy the appendix was found to be gangrenous with localised abscess collection, the tip of the appendix was contained within the right indirect inguinal hernia sac (Figure 2).

The abscess was drained and the appendix was carefully separated from the inguinal canal and excised. The inguinal hernia was not repaired owing to the risk of mesh sepsis and surgical site infection due to the localised abscess collection. An intra-abdominal drain was placed in the pelvis via the suprapubic port site. Within two postoperative days the patient had made an excellent recovery without any complications, the drain was removed and he was discharged on the second postoperative day. During six week follow-up, the patient was asymptomatic with no palpable findings of groin swelling or cough impulse although there was radiological evidence of a small right inguinal hernia in the preoperative period. This was because the hernia was very small and could only be identified first radiologically. Because the patient was asymptomatic during the follow-up, the hernia was not repaired.

Histological examination of the appendix revealed acute ulcerative, suppurative, transmural appendicitis with associated serosal inflammation. Fibrosis of the wall suggested an earlier recent bout of inflammation. There was no evidence of parasitic infestation, histiocytic granuloma formation or neoplasia.

## Discussion

Amyand’s hernia is found almost exclusively in males and usually occurs on the right side [2,8]. Left-sided Amyand’s hernia has been described but is an exceptionally rare occurrence and usually results from bowel malrotation, situs invertus totalis, or a highly mobile caecum [9]. Due to anatomical differences (patency of processus vaginalis) it is three times as common in children [2].

It has been hypothesized that the appendix is vulnerable to trauma when it enters the hernial sac, local adhesions cause the appendix to become incarcerated which could result in changes in the intraluminal pressure causing inflammation of the appendix and subsequent migration of intraluminal microorganisms into the appendix wall [10,11].

CT scanning is able to diagnose Amyand’s hernia preoperatively; the appendix can be visualised within the inguinal canal. Signs of inflammation include increased luminal diameter, fluid collection, peri-appendicular fat stranding and caecal thickening. Ultrasound may also demonstrate the presence of a blind-ending, non-compressible loop within an inguinal hernial sac; inflammation is suspected by finding a dilated appendix lumen (luminal diameter >7.2mm), an increased vascularity of the wall and tenderness on compression [9].

Historically, the management of Amyand’s hernia was based on Losanoff and Basson’s criteria: type 1, normal appendix; type 2, acute appendicitis localised within the sac; type 3, acute appendicitis with peritonitis; type 4, acute appendicitis with other abdominal pathology. They suggested reduction of the appendix and herniorrhaphy for type 1, appendectomy through hernia incision and herniotomy for type 2, and laparotomy with subsequent hernia repair for type 3 and 4 [12,13]. Our case was Losanoff and Basson's type 3. He underwent appendicectomy only but the hernia was not repaired owing to the risk of mesh sepsis and surgical site infection due to the localised abscess collection.

Previously Amyand’s hernia was diagnosed at operation as an incidental finding of an appendix within the hernial sac. There have been only a few publications about preoperative imaging, but with the increased use of CT scanning it is now possible to obtain a preoperative diagnosis and thus plan a laparoscopic approach, alternatively should an Amyand’s hernia be found at laparoscopy it is reasonable to perform a laparoscopic appendicectomy. Due to the rarity of the condition, evidence of successful laparoscopic appendicectomy is limited. Following a successful laparoscopic appendicectomy one could consider an interval hernia repair at a later date to prevent possibility of mesh sepsis and surgical site infection [1,2,7,14,15]. During postoperative follow-up, our patient was asymptomatic with no palpable clinical finding of groin swelling. This was because the hernia was very small despite CT scan in the preoperative period confirming presence of a small groin hernia. Because of the very small nature of the hernia and patient being asymptomatic, a hernia repair was not indicated.

## Conclusions

Due to the increased availability of cross-sectional imaging (CT scan and ultrasound scan), it is now possible to diagnose Amyand’s hernia preoperatively, thus it has become less common to unexpectedly encounter an inflamed appendix during emergency “strangulated” inguinal hernia repair. A preoperative diagnosis permits a laparoscopic approach to be taken and would seem to be beneficial in reducing the incidence of surgical site infection.
